# Identifying key predictors of post-stroke depression and cognitive impairment in acute stroke survivors

**DOI:** 10.3389/fneur.2026.1636511

**Published:** 2026-05-20

**Authors:** Zhiwen Yan, Huan Zhao, Jianjun Chen, Fang Liu, Lei Gong, Yingli Li, Jie Zhang, Mi Xiao, Jun Mu

**Affiliations:** 1 Department of Neurology, The First Affiliated Hospital of Chongqing Medical University, Chongqing, China; 2 Chongqing Key Laboratory of Neurobiology, Institute of Neuroscience, Chongqing Medical University, Chongqing, China

**Keywords:** biomarkers, post-stroke cognitive impairment, post-stroke depression, risk factors, stroke

## Abstract

**Background:**

Post-stroke depression (PSD) and post-stroke cognitive impairment (PSCI) are prevalent complications in aging stroke survivors and are often overlooked due to the lack of early diagnostic indicators, leading to poor prognosis. Identifying reliable predictors is crucial for timely intervention.

**Methods:**

This prospective cohort study followed 78 acute stroke survivors for 6 months. A composite neuropsychological outcome—defined as the development of PSD and/or PSCI—was determined using the Diagnostic and Statistical Manual of Mental Disorders-5th Edition (DSM-5) and NINDS-CSN criteria. To account for the limited sample size, multivariable Firth’s penalized logistic regression was employed to identify independent predictors, generating robust odds ratios (ORs) and 95% confidence intervals (CIs). An exploratory classification and regression tree (CART) analysis was also conducted for hypothesis generation.

**Results:**

The final cohort comprised 78 acute ischemic stroke survivors with a median age of 62 years (IQR 51–71). Among these participants, 26.0% were women, and the median admission score on the National Institutes of Health Stroke Scale (NIHSS) was 3 (IQR 1–5). Within 6 months, 56 patients (71.8%) developed the composite outcome (13 experienced PSCI alone, 24 had PSD alone, and 19 had both conditions). A multivariable analysis revealed that right hemisphere lesions (OR = 9.019, 95% CI: 1.329–61.213, *p* = 0.016), greater baseline emotional distress (higher 9-item Patient Health Questionnaire (PHQ-9) scores; OR = 5.157, 95% CI: 1.835–14.494, *p* < 0.001), and pre-existing cognitive vulnerability (lower Mini–Mental State Examination (MMSE) scores; OR = 0.714, 95% CI: 0.517–0.984, *p* = 0.023) were independent predictors of poor neuropsychological outcomes. Advanced age (*p* = 0.094) and elevated urea levels (*p* = 0.095) showed only marginal trends. Exploratory CART modeling highlighted the hierarchical interaction of these baseline clinical scores for risk stratification.

**Conclusion:**

Right hemisphere lesions, early emotional distress, and baseline cognitive vulnerability independently predicted a high risk of composite neuropsychological impairment at 6 months post-stroke. Rather than serving merely as novel biomarkers, high baseline PHQ-9 scores and low MMSE scores reflected the persistence of early distress and poor cognitive reserve, respectively. These highly accessible clinical parameters facilitate early risk stratification, emphasizing the absolute need for immediate psychological triage and integrated, long-term cognitive-emotional monitoring.

## 1 Introduction

Stroke remains a leading global health challenge. According to the World Stroke Organization (WSO) Global Stroke Fact Sheet 2022, stroke is the second leading cause of death and the third leading cause of death and disability combined worldwide (1). Recent data from the Global Burden of Disease Study 2021 further emphasize that, without effective intervention, the stroke-related burden will escalate significantly through 2050 (2). China, in particular, faces the highest lifelong risk of stroke and the most significant disease burden globally (3), where the prevalence of comorbid dementia is also rising sharply (4). While acute-phase survival rates have improved due to advancements in reperfusion therapies, long-term neuropsychological sequelae—most notably post-stroke depression (PSD) and post-stroke cognitive impairment (PSCI)—have emerged as critical barriers to functional recovery (5). Research indicates that approximately 70% of stroke survivors experience varying degrees of cognitive decline and that 30–80% exhibit depressive symptoms during their recovery phase (6, 7).

The coexistence of PSD and PSCI is not merely coincidental but reflects a complex bidirectional interplay. Clinically, depression can exacerbate cognitive deficits, while significant cognitive impairment often increases the risk of developing depressive symptoms (8, 9). From a pathophysiological perspective, both conditions share overlapping neuroanatomical substrates, such as damage to frontal–subcortical circuits, and common biological pathways, including neuroinflammation, oxidative stress, and white matter integrity disruption (10–14). A study in Pakistan demonstrated that individuals with moderate-to-severe dementia were 16.6 times more likely to experience depression (15). Furthermore, evidence from a large-scale Chinese cohort showed that Mini–Mental State Examination (MMSE) scores were significantly lower in patients with persistent PSD compared to those in remission (8). Given these shared mechanisms and their synergistic negative impact on patient prognosis and quality of life, there is an increasing clinical rationale for investigating PSD and PSCI as a composite neuropsychological outcome—representing the overall “neuropsychological burden” that reflects the global vulnerability of the aging brain to vascular insult (8, 16).

Despite their prevalence, early identification of patients at high risk for these complications remains challenging. Current screening methods often depend on subjective clinical assessments or scales administered after the acute phase, which can lead to missed opportunities for early intervention. While some structural imaging features have been identified, there is a lack of reliable, easily accessible biochemical markers and baseline clinical indicators that can predict long-term psychological and cognitive decline (8, 11). Recent studies have begun to explore the role of metabolic indicators and inflammatory markers, such as the systemic immune-inflammation index and dietary inflammatory potential; however, their predictive value in aging stroke populations requires further validation (16).

Therefore, this prospective study aims to dynamically track acute stroke survivors over a 6-month period to identify key baseline predictors of the development of PSD and/or PSCI. We hypothesize that a combination of baseline neuropsychological scale scores (MMSE and PHQ-9) and specific biochemical indicators—reflecting systemic stress and metabolic status in older adults—can serve as reliable biomarkers for early risk stratification. By identifying these factors, we aim to provide a scientific basis for personalized preventive strategies to improve the long-term prognosis of stroke survivors.

## 2 Methods

### 2.1 Study participants

The study participants were stroke patients admitted to the Department of Neurology, the First Affiliated Hospital of Chongqing Medical University, within 72 h of symptom onset, between October 2018 and June 2022. Baseline data collection included cranial magnetic resonance imaging (MRI), cognitive assessment, and neuropsychological evaluation. This study was approved by the Ethics Committee of the First Affiliated Hospital of Chongqing Medical University (Approval no. 2020–828).

#### 2.1.1 Inclusion and exclusion criteria

The inclusion criteria were as follows:

1. Age between 18 and 80 years.
2. Stroke diagnosis within 72 h of onset, confirmed by clinical presentation and cranial imaging, with the completion of cognitive and neuropsychological assessments within 1 week as part of baseline evaluation.
3. Willingness to participate in scale assessments and follow-up.
4. Provision of informed consent.

The exclusion criteria were as follows:

1. Patients with severe aphasia, impaired consciousness, or inability to cooperate.
2. Patients with severe liver or kidney dysfunction or severe cardiac diseases.
3. Patients with other conditions that may affect cognition or with severe mental illnesses.
4. Withdrawal from follow-up.

**Figure 1:**
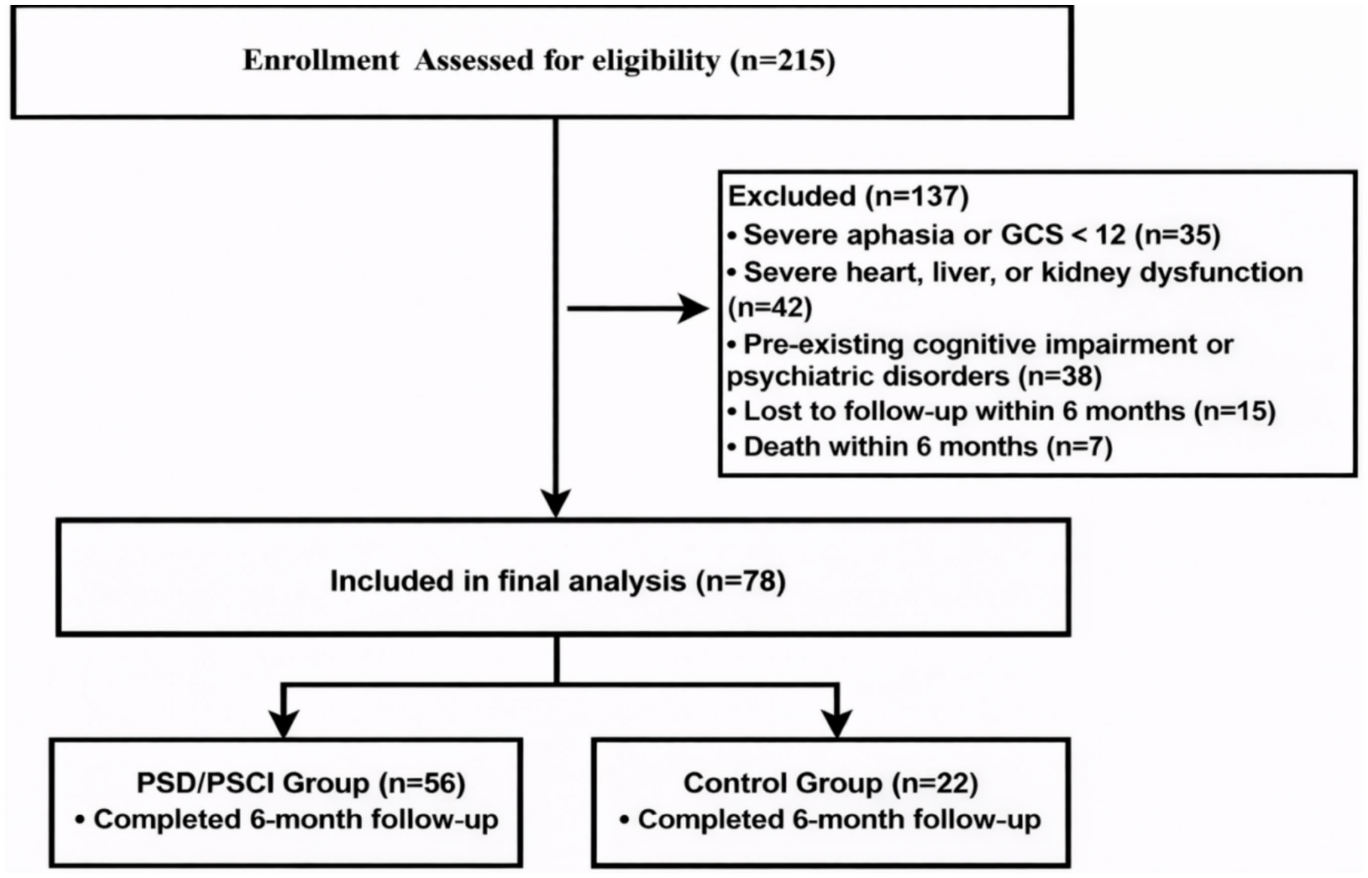
Flowchart of patient recruitment, screening, and group assignment.

The patient recruitment and screening process is illustrated in the flow diagram (Figure 1). Initially, 215 acute stroke patients were screened at our center between October 2018 and June 2022. Following the pre-defined inclusion and exclusion criteria, 137 patients were excluded. The primary reasons for exclusion included the following: 35 patients with severe aphasia or impaired consciousness precluding assessment, 42 patients with severe systemic diseases (e.g., end-stage renal disease or NYHA Class III/IV heart failure), 38 patients with pre-existing cognitive impairment or psychiatric disorders, and 22 patients who withdrew or died during the 6-month follow-up period. Consequently, a final cohort of 78 patients was included in the analysis.

#### 2.1.2 Diagnostic criteria and study grouping

The diagnostic criteria used in this study, although based on the Chinese Expert Consensus, are strictly aligned with international standards to ensure reproducibility. Specifically, the diagnosis of PSD followed the symptom criteria for depressive disorders as outlined in the *Diagnostic and Statistical Manual of Mental Disorders*-5th Edition (DSM-5), with a 9-item Patient Health Questionnaire (PHQ-9) score of ≥ 5 used as a screening threshold, followed by confirmation through a clinical interview. Similarly, PSCI was defined according to the NINDS-CSN guidelines for vascular cognitive impairment, characterized by a decline in at least one cognitive domain (assessed using the MMSE and MoCA) that occurred after stroke and persisted beyond the acute phase (17–19).

Based on the 6-month follow-up assessments, the final cohort was categorized into two groups:

1. Stroke-related depression/cognitive impairment group.
2. Non-PSCI/PSD group: Stroke survivors without depression/cognitive impairment.

### 2.2 Research methods

#### 2.2.1 Clinical data

We employed structured questionnaires to collect general information about patients. This included age, sex, education level, occupation, smoking history, alcohol consumption history, exercise habits, dietary habits, presence of comorbidities (such as atrial fibrillation, hypertension, coronary artery disease, diabetes, and hyperlipidemia), prior stroke history, family history, NIHSS score, lesion location, presence of large vessel stenosis, TOAST classification, and risk factors for pneumonia. Relevant laboratory test indicators were also collected.

#### 2.2.2 Scale assessment

Two trained neurologists conducted structured interviews and scale assessments of the patients at 1 week, 1 month, 3 months, and 6 months after the onset of symptoms. The primary outcome was the occurrence of PSD and/or PSCI. The assessment tools used included the modified Rankin Scale (mRS), the Glasgow Coma Scale (GCS), the National Institutes of Health Stroke Scale (NIHSS), the Mini–Mental State Examination (MMSE), the PHQ-9, the 7-item Generalized Anxiety Disorder (GAD-7), the Athens Insomnia Scale (AIS), and the MoCA. The PHQ-9 was utilized due to its established validity and reliability as a brief measure of depression severity (20), as well as its proven accuracy in screening post-stroke depression in stroke survivors (19).

#### 2.2.3 Nutritional screening tools

The Controlling Nutritional Status (CONUT) scoring system (21) was used to estimate nutritional status. It uses serum albumin levels, lymphocyte count, and total cholesterol levels. The CONUT scores are categorized into four levels of malnutrition: Normal (0–1), mild (2–4), moderate (5–8), and severe (9–12). The Prognostic Nutritional Index (PNI) is calculated using the following formula: PNI = serum albumin concentration (g/L) + 5 * lymphocyte count (10^9^/L). Based on the PNI score, individuals are categorized as normal (≥50), slightly malnourished (45 ≤ PNI < 50), moderately malnourished (40 ≤ PNI < 45), and severely malnourished (< 40).

#### 2.2.4 Specimen collection and blood index determination

Venous blood samples were collected from all patients within 24 h of admission after an overnight fast. Serum was obtained by centrifugation at 3000 rpm for 10 min at 4 °C. Laboratory indicators, including complete blood count, liver function, renal function, and lipid profiles, were measured using an automated biochemical analyzer in accordance with standard laboratory protocols.

### 2.3 Statistical analysis

Statistical analysis was performed using SPSS version 19.0 (IBM Corp., Armonk, NY, USA) and R software (version 4.4.3). The normality of continuous variables was assessed using the Kolmogorov–Smirnov test. Normally distributed data were expressed as mean ± standard deviation (SD) and compared using the independent samples *t*-test or one-way analysis of variance (ANOVA). Non-normally distributed data were presented as median (interquartile range, IQR) and analyzed using the Mann–Whitney U test or Kruskal–Wallis test. Categorical variables were expressed as frequencies (percentages) and analyzed using the chi-squared test or Fisher’s exact test.

To identify independent predictors of PSD/PSCI, a multivariable model was constructed using Firth’s penalized logistic regression. This method was specifically selected to provide robust estimations and reduce parameter bias inherent in small sample sizes or sparse categorical data (22). Following established statistical recommendations to prevent the omission of potential confounders, variables with a *p* < 0.10 in the univariate analysis were entered into the multivariable model (23, 24). Furthermore, “lesion side” (*p* = 0.104 in univariate analysis) was pre-specified for inclusion based on its well-documented clinical significance in post-stroke neuropsychological outcomes (25), prioritizing a theory-driven selection approach over a purely data-driven selection approach.

A two-tailed *p*-value of < 0.05 was considered statistically significant. As a secondary exploratory analysis, the classification and regression tree (CART) was utilized to investigate potential hierarchical relationships and generate clinical risk profiles based on the identified independent factors. Given the limited sample size, the CART model was primarily used for hypothesis generation rather than definitive risk stratification.

## 3 Results

### 3.1 Study population and baseline characteristics

The participant recruitment process is shown in the flowchart (Figure 1). Of the 215 patients initially screened, 78 acute stroke survivors were included in the final analysis. To enhance statistical robustness and focus on the overall neuropsychological burden, patients were categorized into two groups based on a composite outcome: PSD/PSCI group (n = 56, 71.8%) and control group (n = 22, 28.2%). The affected group comprised patients with PSCI alone (16.7%), PSD alone (30.8%), and comorbid PSD/PSCI (24.4%). Detailed subgroup data are provided in Supplementary Tables S1–S4.

Baseline clinical characteristics are presented in Table 1. Patients who developed PSD/PSCI were generally older than those in the control group. The comorbidity burden was notably higher in the affected group; specifically, 84% of patients in the PSD/PSCI group had ≥ 2 chronic conditions, compared to 68% in the control group. A history of prior stroke and coronary heart disease also showed higher prevalence in the affected group, reaching near statistical significance.

**Table 1:** Baseline demographic and clinical characteristics of the study population.

Characteristic	Overall	Control	PSD/PSCI	*P*-value
	*N* = 78	*N* = 22	*N* = 56	
Age (years)	62 (51, 71)	57 (51, 65)	65 (52, 72)	0.058
Female sex	20 (26%)	5 (23%)	15 (27%)	0.712
Education (years)	12 (9, 15)	12 (9, 15)	12 (9, 15)	0.883
Comorbidity burden (≥ 2 conditions)	62 (79%)	15 (68%)	47 (84%)	0.133
Hypertension	62 (79%)	15 (68%)	47 (84%)	0.133
Hypertensive heart disease	21 (27%)	5 (23%)	16 (29%)	0.601
Diabetes mellitus	34 (44%)	9 (41%)	25 (45%)	0.765
Hyperlipidemia	39 (50%)	10 (45%)	29 (52%)	0.615
Coronary heart disease	10 (13%)	0 (0%)	10 (18%)	0.054
Large-vessel stenosis	42 (54%)	11 (50%)	31 (55%)	0.669
Smoking	37 (47%)	12 (55%)	25 (45%)	0.431
Drinking	33 (42%)	11 (50%)	22 (39%)	0.389
Exercise	42 (54%)	14 (64%)	28 (50%)	0.277
Lesion site				0.104
Left	28 (36%)	11 (50%)	17 (30%)	
Right	50 (64%)	11 (50%)	39 (70%)	

Values are expressed as *n* (%) for categorical variables and median (interquartile range, IQR) for continuous variables. *P*-values were calculated using Fisher’s exact test for categorical variables and the Mann–Whitney U test for continuous variables. Variables with a *p* < 0.15 in the univariate analysis were considered candidates for multivariable regression.

### 3.2 Laboratory and nutritional assessment

The comparison of laboratory indicators (Table 2) revealed that specific metabolic and inflammatory markers were significantly associated with poor outcomes. Urea levels were significantly higher in the PSD/PSCI group compared to the control group (5.64 ± 1.56 vs. 4.67 ± 0.89 mmol/L, *p* = 0.004). Similarly, LDH levels were significantly elevated in the affected group (237.02 vs. 168.62 U/L, *p* = 0.016).

**Table 2:** Comparison of admission laboratory indicators between the control and PSD/PSCI groups.

Characteristic	Overall	Control	PSD/PSCI	*P*-value
	*N* = 78	*N* = 22	*N* = 56	
WBC (10^9/L)	7.77 (2.46)	8.29 (2.84)	7.57 (2.29)	0.345
Hb (g/L)	142 (16)	143 (17)	142 (16)	0.505
PLT (10^9/L)	208 (60)	228 (57)	201 (60)	0.064
RDW	14.29 (11.27)	12.99 (1.45)	14.80 (13.26)	0.16
N count	5.43 (2.28)	5.69 (2.61)	5.32 (2.16)	0.735
L count	1.60 (0.55)	1.59 (0.49)	1.60 (0.58)	0.894
CRP	14 (50)	4 (3)	18 (59)	0.447
PCT	0.100 (0.490)	0.050 (0.044)	0.120 (0.577)	0.795
ESR	23 (19)	22 (16)	24 (21)	0.991
INR	0.95 (0.12)	0.97 (0.07)	0.94 (0.13)	0.802
Albumin (g/L)	42 (4)	42 (4)	41 (4)	0.776
AST	26 (7)	24 (4)	27 (8)	0.081
ALT	28 (13)	24 (8)	29 (15)	0.18
LDH	218 (114)	171 (43)	237 (127)	0.073
TBIL	12.3 (5.9)	11.2 (4.3)	12.7 (6.4)	0.289
DBIL	2.56 (1.85)	1.81 (1.15)	2.86 (2.00)	0.037
IBIL	11.1 (12.5)	9.4 (4.1)	11.8 (14.6)	0.798
Cr	71 (25)	77 (25)	69 (24)	0.096
Urea (mmol/L)	5.36 (1.46)	4.67 (0.89)	5.64 (1.56)	0.005
UA	348 (105)	359 (97)	344 (109)	0.334
TC	4.65 (1.05)	4.79 (0.98)	4.59 (1.07)	0.665
TG	1.97 (1.61)	2.42 (1.69)	1.79 (1.56)	0.137
HDL-C	1.11 (0.24)	1.09 (0.23)	1.12 (0.24)	0.743
LDL-C	2.90 (0.94)	2.85 (0.85)	2.92 (0.98)	0.689
HCY	18 (12)	20 (17)	17 (10)	0.613
FA	11 (7)	9 (5)	12 (7)	0.135
VB12	277 (140)	246 (99)	289 (152)	0.286

Values are presented as mean (standard deviation, SD). *P*-values were derived from the independent samples t-test or the Mann–Whitney U test, as appropriate. WBC, white blood cell; Hb, hemoglobin; PLT, platelet; RDW, red cell distribution width; CRP, C-reactive protein; PCT, procalcitonin; ESR, erythrocyte sedimentation rate; INR, international normalized ratio; AST, aspartate aminotransferase; ALT, alanine aminotransferase; LDH, lactate dehydrogenase; TBIL, total bilirubin; DBIL, direct bilirubin; IBIL, indirect bilirubin; Cr, creatinine; Urea, blood urea nitrogen; UA, uric acid; TC, total cholesterol; TG, triglyceride; HDL-C, high-density lipoprotein cholesterol; LDL-C, low-density lipoprotein cholesterol; HCY, homocysteine; FA, folic acid; VB12, vitamin B12.

Nutritional assessment (Table 3) indicated that the PSD/PSCI group had a significantly higher nutritional risk. According to the CONUT criteria, 55% of the affected group were classified as at nutritional risk (score ≥ 2), whereas only 27% of the control group met this threshold (*p* = 0.025).

**Table 3:** Nutritional status and dietary patterns of stroke patients.

Characteristic	Overall	Control	PSD/PSCI	*P*-value
	*N* = 78	*N* = 22	*N* = 56	
High-fat diet	42 (54%)	13 (59%)	29 (52%)	0.56
High-fiber diet	13 (17%)	1 (4.5%)	12 (21%)	0.096
CONUT Score				0.194
0	13 (17%)	4 (18%)	9 (16%)	
1	28 (36%)	12 (55%)	16 (29%)	
2	20 (26%)	2 (9.1%)	18 (32%)	
3	11 (14%)	3 (14%)	8 (14%)	
4	5 (6.4%)	1 (4.5%)	4 (7.1%)	
5	1 (1.3%)	0 (0%)	1 (1.8%)	
Nutritional Risk (CONUT ≥ 2)	37 (47%)	6 (27%)	31 (55%)	0.025
PNI Score	43 (41, 45)	43 (40, 47)	43 (41, 45)	0.942

Data are presented as median (IQR) or *n* (%). *P-*values were calculated using the Mann–Whitney U test. NIHSS, National Institutes of Health Stroke Scale; mRS, modified Rankin Scale; ADL, Activities of Daily Living; MMSE, Mini–Mental State Examination; PHQ-9, 9-item Patient Health Questionnaire; GAD-7, 7-item Generalized Anxiety Disorder scale; AIS, Athens Insomnia Scale.

### 3.3 Baseline clinical and neuropsychological scales

At baseline (within the first week after stroke onset), patients in the PSD/PSCI group exhibited significantly higher emotional distress and sleep disturbances (Table 4). Median scores were significantly higher for the PHQ-9 (5 vs. 1, *p* < 0.001), GAD-7 (2 vs. 0, *p* = 0.012), and AIS (3 vs. 0, *p* = 0.003). Cognitive vulnerability was also evident, with the PSD/PSCI group demonstrating significantly lower baseline MMSE scores (median 26 vs. 28, *p* = 0.018) and higher ADL scores (median 22.50 vs. 14.00, *p* = 0.015), indicating reduced functional independence at baseline.

**Table 4:** Baseline clinical and neuropsychological scale scores.

Characteristic	Overall	Control	PSD/PSCI	*P*-value
	*N* = 78	*N* = 22	*N* = 56	
NIHSS	3 (1, 5)	3 (1, 3)	3 (1, 6)	0.277
mRS				0.467
1	46 (59%)	15 (68%)	31 (55%)	
2	12 (15%)	3 (14%)	9 (16%)	
3	9 (12%)	3 (14%)	6 (11%)	
4	11 (14%)	1 (4.5%)	10 (18%)	
ADL	20 (14, 34)	14 (14, 24)	24 (16, 35)	0.012
Baseline MMSE	27 (25, 29)	28 (27, 30)	26 (25, 29)	0.037
Baseline PHQ-9	3 (1, 7)	1 (0, 1)	5 (2, 9)	< 0.001
Baseline GAD-7	1 (0, 3)	0 (0, 1)	2 (0, 6)	0.004
Baseline AIS score	2 (0, 5)	0 (0, 2)	3 (1, 6)	0.002
Pneumonia Risk	4 (2, 7)	3 (1, 5)	5 (2, 7)	0.1

Values are expressed as *n* (%) or mean (SD). Nutritional risk was defined as a CONUT score of ≥ 2. CONUT, Controlling Nutritional Status; PNI, Prognostic Nutritional Index. *P*-values were calculated using Fisher’s exact test for dietary habits and nutritional risk and the independent samples *t*-test for the PNI score.

### 3.4 Univariate predictors of the composite outcome (Forest plot)

**Figure 2:**
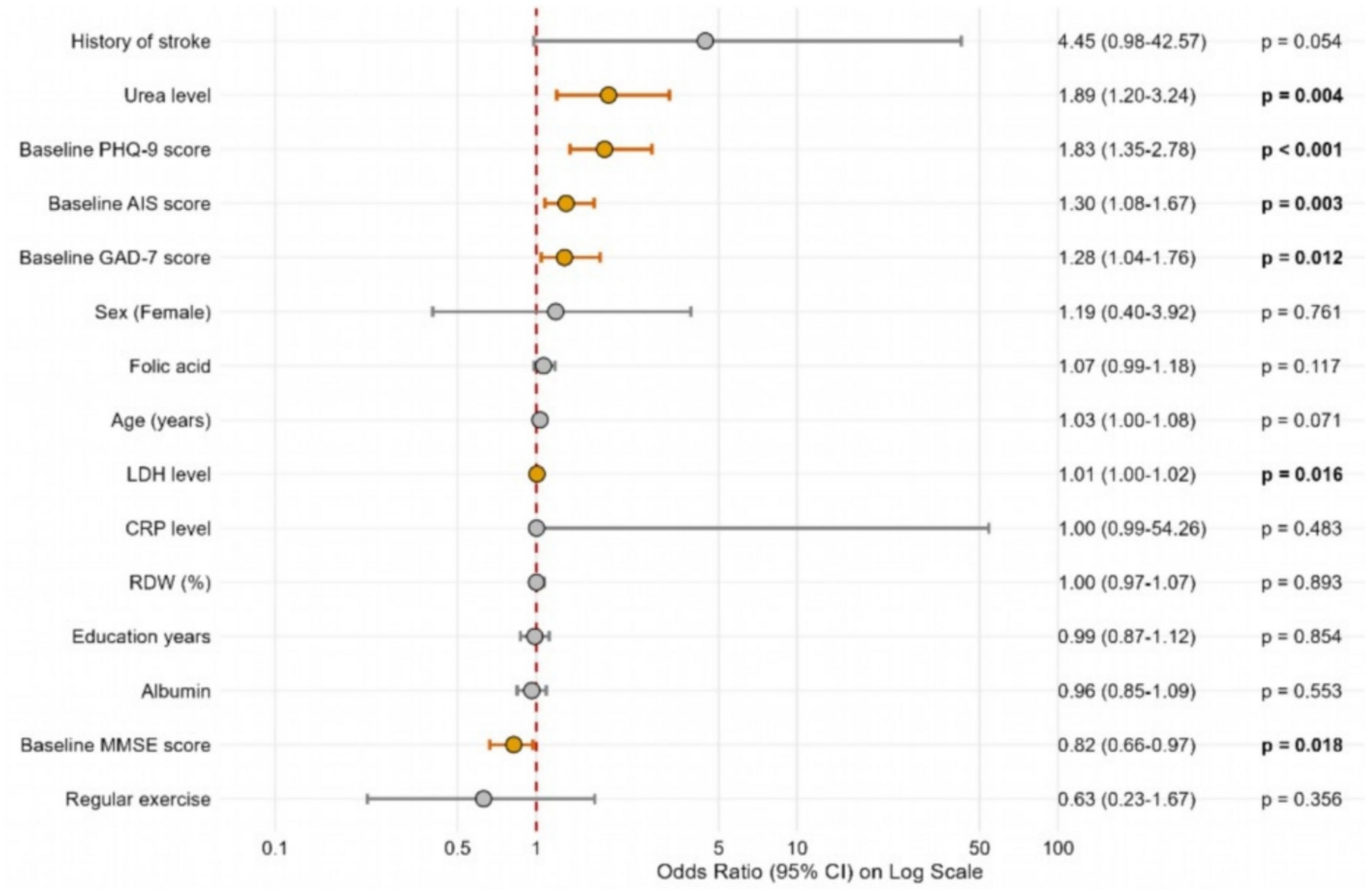
Forest plot of univariate predictors for the composite neuropsychological outcome.

Note: Odds ratios (ORs), 95% confidence intervals (CIs), and *p*-values were derived from univariate Firth’s penalized logistic regression to account for sparse categorical data. Significant predictors (*p* < 0.05) are highlighted in orange.

As shown in the forest plot (Figure 2), higher baseline PHQ-9 scores (odds ratio (OR) = 1.83, 95% confidence interval (CI): 1.35–2.78, *p* < 0.001), urea levels (OR = 1.89, *p* = 0.004), and baseline AIS scores (OR = 1.30, *p* = 0.003) were the strongest risk factors. Conversely, higher baseline MMSE scores (OR = 0.82, *p* = 0.018) served as a significant protective factor. A history of prior stroke (OR = 4.45, *p* = 0.054) and advanced age (*p* = 0.071) exhibited strong trends as risk factors.

### 3.5 Independent predictors in multivariable analysis

Variables with a *p* < 0.10 in the univariate analysis were entered into a multivariable Firth penalized logistic regression model (Table 5). The analysis identified right hemisphere lesions (OR = 9.02, 95% CI: 1.33–61.21, *p* = 0.016), higher baseline PHQ-9 scores (OR = 5.16, 95% CI: 1.84–14.49, *p* < 0.001), and lower baseline MMSE scores (OR = 0.71, 95% CI: 0.52–0.98, *p* = 0.023) as independent predictors of the 6-month composite neuropsychological outcome.

**Table 5:** Multivariable firth’s penalized logistic regression analysis of independent predictors for the composite neuropsychological outcome (PSD/PSCI).

Variable	Right hemisphere lesion, *n* (%)	Age	Baseline PHQ-9	Baseline MMSE	Urea (mmol/L)
P	0.016	0.094	< 0.001	0.023	0.095
OR (95% CI)	9.019 (1.329–61.213)	1.075 (0.991–1.167)	5.157 (1.835–14.494)	0.714 (0.517–0.984)	1.432 (0.898–2.285)

OR, Odds Ratio; CI, Confidence Interval; PHQ-9, 9-item Patient Health Questionnaire; MMSE, Mini–Mental State Examination.

### 3.6 Subgroup analysis in the PSD/PSCI group

**Figure 3:**
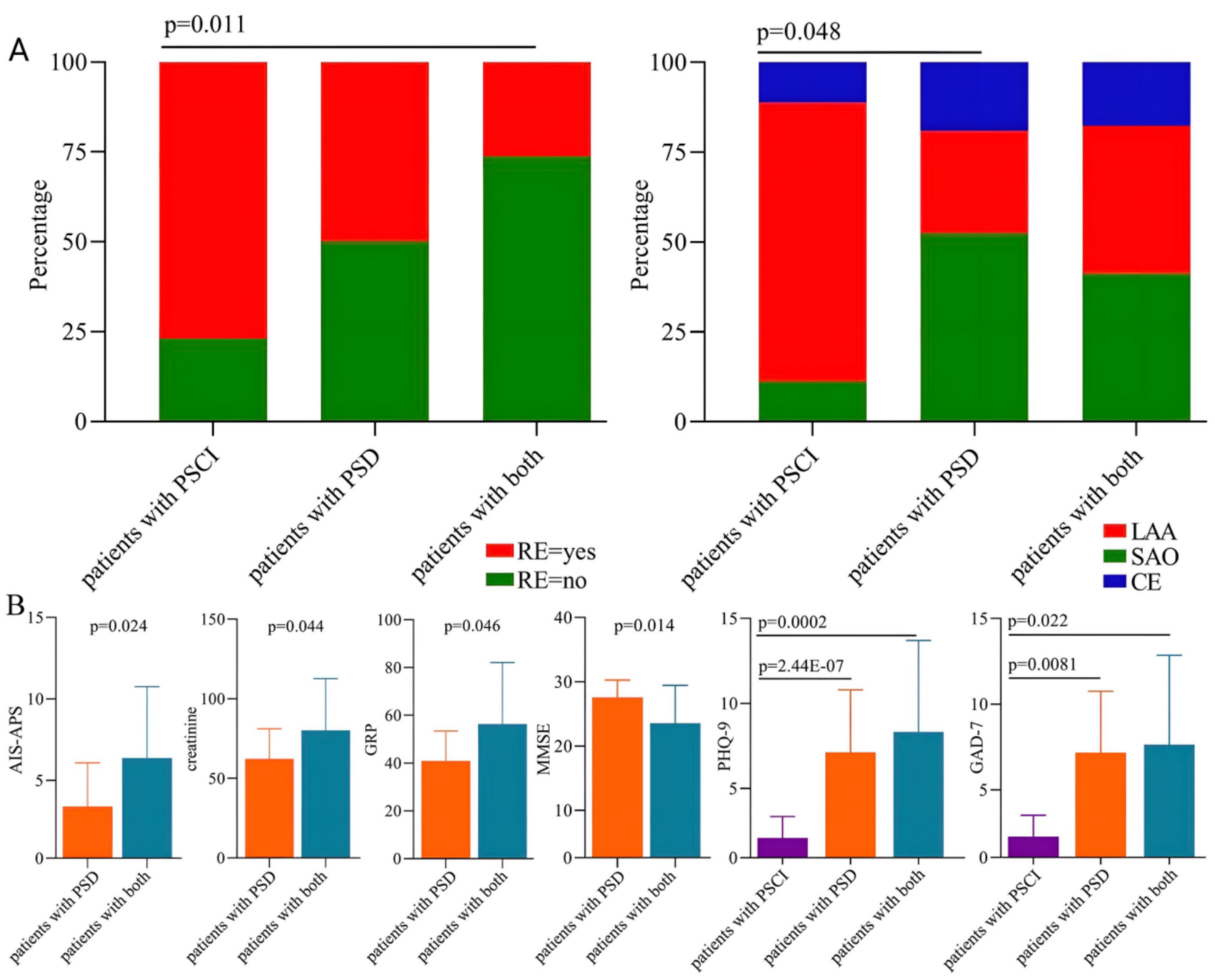
Subgroup analysis in patients with PSD/PSCI. **(A)** Two categorical variables differed significantly among the three subgroups: Regular exercise and TOAST classification. **(B)** Six continuous variables differed significantly among the three subgroups: PHQ-9, GAD-7, MMSE, AIS-APS, Pro-GRP, and creatinine. Pro-GRP, pro-gastrin-releasing-peptide.

To explore whether there were significant differences in these variables among subgroups of patients with PSD/PSCI, a subgroup analysis was conducted. The results showed that compared to patients with both PSD and PSCI, patients with PSCI alone had a significantly higher level of regular exercise (*p* = 0.011) (Figure 3A). The subgroup analysis also found that the percentage of three classifications was significantly different between patients with PSCI (small artery occlusion [SAO], 11.11%; large artery atherosclerosis [LAA], 77.78%; and cardioembolism [CE], 11.11%) and those with PSD (SAO, 52.38%; LAA, 28.57%; and CE, 19.05%) (*p* = 0.048, Figure 3A).

Furthermore, the subgroup analysis showed that compared to patients with PSD alone, patients with comorbid PSD and PSCI had significantly higher AIS-APS scores (*p* = 0.024), creatinine levels (*p* = 0.044), and pro-gastrin-releasing-peptide (Pro-GRP) levels (*p* = 0.046), along with lower MMSE scores (*p* = 0.014) (Figure 3B). We also found that both PHQ-9 scores (*p* < 0.001) and GAD-7 scores (*p* = 0.0081) were significantly higher in patients with PSD compared to those with PSCI. Compared to patients with PSCI alone, patients with both PSD and PSCI also had significantly higher PHQ-9 (*p* = 0.0002) and GAD-7 scores (*p* = 0.022) (Figure 3B).

### 3.7 Exploratory analysis: hierarchical risk profiles using the CART

**Figure 4:**
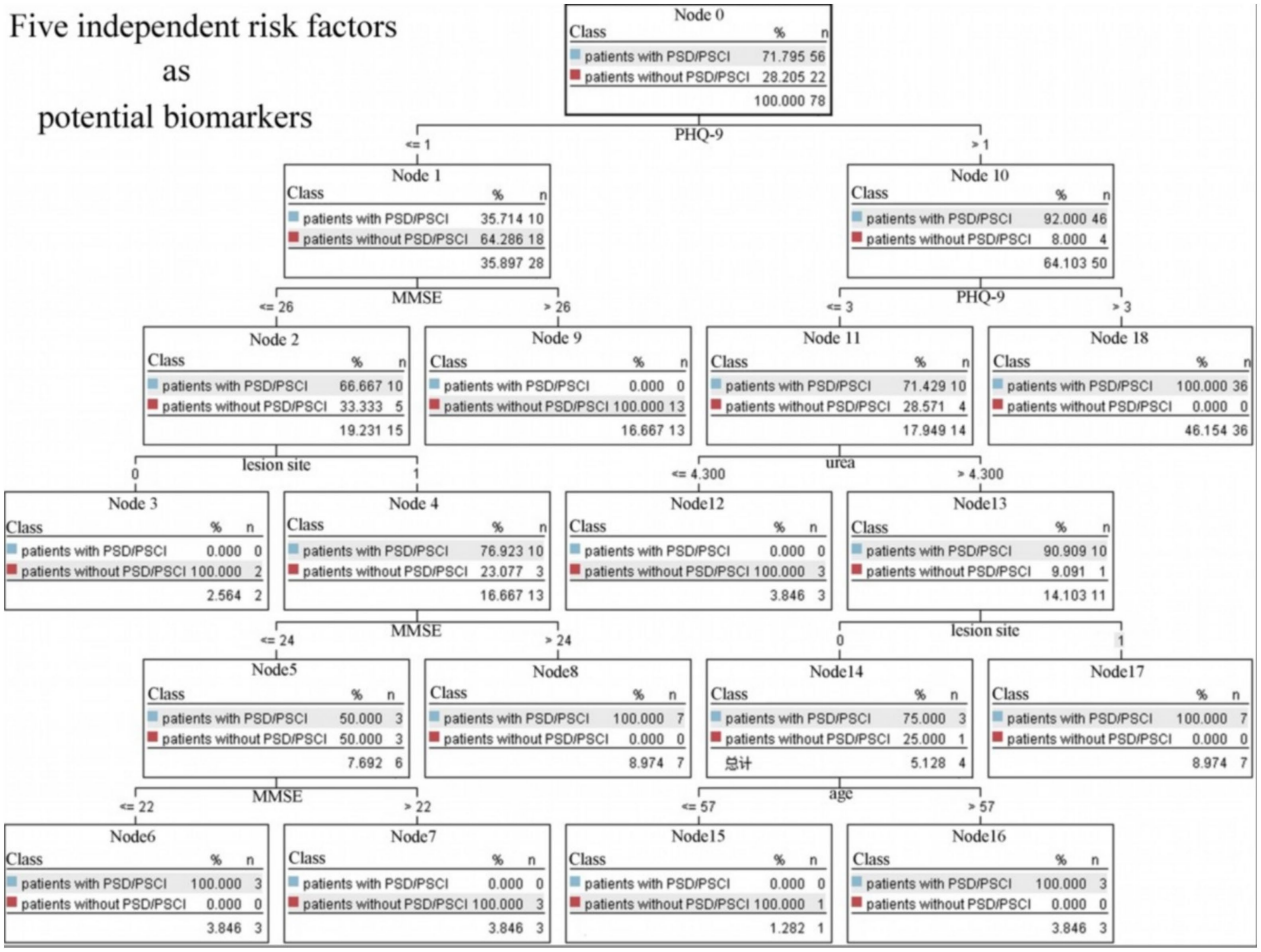
Five independent risk factors as potential biomarkers for the diagnosis of PSD/PSCI.

To visualize potential hierarchical relationships among predictors, an exploratory CART analysis was conducted (Figure 4). While the model achieved high classification accuracy within this cohort, we acknowledge that this result may reflect overfitting due to the small sample size. The model identified the baseline PHQ-9 score as the primary splitting node for risk stratification. For patients with low baseline emotional distress levels (PHQ-9 ≤ 3), the integration of urea levels and MMSE scores provided additional risk profiles, suggesting that metabolic stress and pre-existing cognitive vulnerability further modulate the risk of post-stroke neuropsychological decline.

## 4 Discussion

Stroke remains the leading cause of disability and death among the Chinese population, with long-term neuropsychological sequelae, specifically PSD and PSCI, posing significant barriers to rehabilitation (6, 7). A critical challenge in clinical practice is the early identification of high-risk patients. In this study, we utilized a composite neuropsychological outcome (PSD and/or PSCI) because these conditions frequently co-occur and share common pathophysiological mechanisms, such as damage to frontal–subcortical circuits and neuroinflammation (15, 26). This integrated clinical approach is supported by recent evidence suggesting that aging neural tissues rely on vascular and metabolic integrity to maintain cognitive and emotional health (13).

The impact of lesion laterality on neuropsychological outcomes remains a subject of intense debate. This study found that patients with right hemisphere lesions are more likely to develop the composite outcome of PSD/PSCI. This aligns with the meta-analysis by Wei et al. (27) but contrasts with other studies suggesting a higher risk following left-sided stroke (28) or reporting no significant association (29). Our findings suggest that damage to the right hemisphere may disrupt non-verbal emotional processing and visuospatial cognition. Interestingly, while lesion side showed only a marginal trend in univariate analysis, its independent predictive value emerged in the multivariable model after adjusting for baseline cognitive and emotional scores. This suggests that the impact of lesion laterality may be masked by other clinical variables, a phenomenon that warrants further investigation in larger cohorts. Furthermore, recent research has linked pro-inflammatory states to reductions in brain volume and the progression of small vessel disease, which further exacerbates the neuropsychological burden regardless of the primary lesion side (30). Damage to critical areas such as the basal ganglia and internal capsule has been linked to the CHANGE risk score, which serves as a reliable tool for screening PSCI risk (31).

In this cohort, elevated urea levels showed a visible trend but did not achieve statistical significance in multivariable analysis (*p* = 0.095). Although traditionally viewed as a marker of renal function, elevated urea levels in acute stroke may reflect a complex interaction between the kidneys and the brain, often referred to as the “kidney–brain axis.” This axis represents a bidirectional communication system where renal function and brain health are interconnected, potentially exacerbated by acute metabolic stress (32). Elevated urea levels might, as a hypothesis, reflect metabolic changes related to stress, which could influence both renal function and neuroendocrine responses, possibly involving activation of the hypothalamic–pituitary–adrenal (HPA) axis (33). However, this trend requires cautious interpretation. Given the lack of statistical significance in this study, these metabolic observations should be considered preliminary and require validation in larger studies to determine their robust predictive value.

Although the number of comorbidities was significant in the univariate analysis, it was not included in the multivariable model due to its potential collinearity with other clinical variables, as well as its less clear direct relationship with the neuropsychological outcomes we sought to predict. We believe that the variables ultimately selected for the multivariable model offer more reliable insights into the key predictors of PSD/PSCI, but this aspect warrants further exploration in future studies.

Similarly, while advanced age did not reach multivariable significance (*p* = 0.094), it demonstrated a numerical trend in our model. The cumulative burden of hypertension and coronary heart disease likely compromises cerebral microcirculation, potentially contributing to the overall neuropsychological burden. In addition, the analysis of baseline scale scores indicated that higher PHQ-9 and lower MMSE scores remain the most reliable independent indicators of future chronic decline (Table 4), emphasizing the need for early neuropsychological screening.

Furthermore, our study highlights the importance of nutritional status. Existing studies have shown that malnutrition is associated with the occurrence of many diseases, and objective screening tools such as the CONUT and PNI have been validated for their prognostic value in various clinical settings (34). In our cohort, while the absolute CONUT score did not show statistical significance (*p* = 0.168), categorization into “nutritional risk” revealed a strong correlation with PSD/PSCI occurrence (*p* = 0.029, Table 3). This is consistent with research by Gu et al. (35), which demonstrated that nutritional risk is associated with higher PSD incidence. Malnutrition may influence brain health via the gut–microbiota–brain axis, a process closely linked to the Dietary Inflammatory Index (DII) (36). Moreover, the structure and activity of gut microbiota are influenced by dietary patterns (37). Specifically, high-fiber diets promote a favorable shift in gut microbiota toward species that produce short-chain fatty acids (SCFAs), which reduce systemic inflammatory responses and promote neural repair (38–40).

Finally, physical activity was identified as a protective factor. Regular exercise likely enhances neurogenesis and modulates gut microbiome diversity (41, 42). Our results are consistent with recent dose–response analyses showing that higher levels of daily movement significantly mitigate cognitive impairment risk in aging populations (43).

We acknowledge several limitations, including the relatively small sample size (*n* = 78), which may increase the risk of model overfitting. To address this, we utilized forward stepwise Firth’s penalized logistic regression and focused on clinically relevant variables. Future large-scale, multicenter cohort studies are required to validate these potential biomarkers.

In addition, we recognize that the exclusion of 15 patients who were lost to follow-up and 7 patients who died within 6 months may have introduced bias. The excluded patients, particularly those who died, were likely to be the most severely ill and at the highest risk for the outcomes under study. As a result, their exclusion may lead to an underestimation of the true severity or progression of the condition, particularly regarding mortality and other adverse outcomes. The loss of these data could lead to a more favorable interpretation of the disease course than is actually accurate. Moreover, patient withdrawal from follow-up may reflect a subgroup with different characteristics, such as greater emotional distress or lower engagement with treatment, which could potentially affect the study’s results.

Given these potential sources of bias, we caution that our findings be interpreted while considering these limitations. Future research should aim to minimize loss to follow-up and include data from patients who experience adverse outcomes, such as death, to better capture the full range of disease progression and outcomes.

## 5 Conclusion

This study demonstrates that right hemisphere lesions, high baseline depressive symptoms (PHQ-9), and pre-existing cognitive vulnerability (MMSE) are critical independent predictors of poor neuropsychological outcomes 6 months after stroke. The association with elevated urea levels suggests a potential “kidney–brain axis” interaction, where acute metabolic stress and HPA axis activation may compromise neural recovery. While moderate-intensity exercise appeared to be a protective factor, variables such as advanced age and nutritional risk showed only preliminary trends and did not reach statistical significance or independence in this small cohort. Moreover, the exclusion of 15 patients lost to follow-up and 7 who died within 6 months could have introduced bias, as these patients were likely the most severely ill, potentially underestimating the true severity of outcomes. Given the relatively small sample size, these findings should be viewed as preliminary biomarkers, with the need for validation in larger, multicenter prospective cohorts to confirm their predictive value and to optimize personalized intervention strategies for aging stroke survivors.

## Data Availability

The raw data supporting the conclusions of this article will be made available by the authors, without undue reservation.

## References

[1] Feigin VL Brainin M Norrving B Martins S Sacco RL Hacke W . Global stroke fact sheet 2022. Int J Stroke. (2022) 17:18–29. doi: 10.1177/17474930211065917 34986727

[2] Naghavi M Ong KL Aali A Ababneh HS. Global burden of 288 causes of death and life expectancy decomposition in 204 countries and territories and 811 subnational locations, 1990-2021: a systematic analysis for the global burden of disease study 2021. Lancet. (2024) 403:2100–32. doi: 10.1016/S0140-673.(24)00367-2, 38582094 PMC11126520

[3] Zhou M Wang H Zeng X Yin P Zhu J Chen W . Mortality, morbidity, and risk factors in China and its provinces, 1990-2017: a systematic analysis for the global burden of disease study 2017. Lancet. (2019) 394:1145–58. doi: 10.1016/S0140-6736(19)30427-1, 31248666 PMC6891889

[4] Jia L Quan M Fu Y Zhao T Li Y Wei C . Dementia in China: epidemiology, clinical management, and research advances. Lancet Neurol. (2020) 19:81–92. doi: 10.1016/s1474-4422(19)30290-x, 31494009

[5] Pinho PJMR Trombin Marques M Sadala Reges D Rocha E. Abstract WP62: post-stroke cognitive impairment and depression in a Brazilian nationally representative sample. Stroke. (2025) 56:1–12. doi: 10.1161/str.56.suppl_1.WP62

[6] Robinson RG Jorge RE. Post-stroke depression: a review. Am J Psychiatry. (2016) 173:221–31. doi: 10.1176/appi.ajp.2015.15030363, 26684921

[7] Huang YY Chen SD Leng XY Kuo K Wang ZT Cui M . Post-stroke cognitive impairment: epidemiology, risk factors, and management. J Alzheimer's Dis. (2022) 86:983–99. doi: 10.3233/jad-215644, 35147548

[8] Zheng MR Chen P Feng Y Zhang Q Su Z Cheung T . Prevalence of depression and cognitive impairment and their inter-relationship and association with quality of life among older stroke survivors: the findings of a national survey in China. Stroke Vasc Neurol. (2025) 11:3623. doi: 10.1136/svn-2024-003623, 40623732 PMC13019049

[9] Chau JPC Lo SHS Zhao J Choi KC Butt L Lau AYL . Prevalence of post-stroke cognitive impairment and associated risk factors in Chinese stroke survivors. J Neurol Sci. (2023) 455:122805. doi: 10.1016/j.jns.2023.122805, 37995462

[10] Mu J Li J. Analysis of radiological features in patients with post-stroke depression and cognitive impairment. Rev Neurosci. (2024) 35:565–73. doi: 10.1515/revneuro-2023-0120, 38417835

[11] Jaiswal V Ang SP Suresh V Nasir Y Joshi A Kumar D . Abstract TP24: c-reactive protein, and homocysteine as a predictors of post stroke depression among stroke patients: a meta-analysis and meta regression. Stroke. (2024) 55:1–12. doi: 10.1161/str.55.suppl_1.TP24

[12] Wen L Yan C Si T Huang L Nie Y Shen H . The predictive role of early inflammation and oxidative stress and the dynamics of cytokines networks in post-stroke depression. J Affect Disord. (2024) 347:469–76. doi: 10.1016/j.jad.2023.12.012, 38065474

[13] Montine TJ Bukhari SA White LR. Cognitive impairment in older adults and therapeutic strategies. Pharmacol Rev. (2021) 73:152–62. doi: 10.1124/pharmrev.120.000031, 33298513 PMC7736830

[14] Ferrucci L Fabbri E. Inflammageing: chronic inflammation in ageing, cardiovascular disease, and frailty. Nat Rev Cardiol. (2018) 15:505–22. doi: 10.1038/s41569-018-0064-2, 30065258 PMC6146930

[15] Huang J Zhou FC Guan B Zhang N Wang A Yu P . Predictors of remission of early-onset poststroke depression and the interaction between depression and cognition during follow-up. Front Psych. (2018) 9:738. doi: 10.3389/fpsyt.2018.00738, 30670990 PMC6331416

[16] Su X Chi F You J Zhao J Wang S Zhou X . Association between systemic immune-inflammation index at admission and post-stroke depression in patients with acute ischemic stroke. Front Neurol. (2025) 16:1686621. doi: 10.3389/fneur.2025.1686621, 41458124 PMC12740874

[17] Wang QDK Yu J . Expert consensus on the management of post-stroke cognitive impairment in 2021. Chin J Stroke. (2021) 16:376–89.

[18] Zhao FY Yue YY Li L Lang SY Wang MW Du XD . Clinical practice guidelines for post-stroke depression in China. Braz J Psychiatry. (2018) 40:325–334. doi: 10.1590/1516-4446-2017-2343 29412338 PMC6899404

[19] Dajpratham P Pukrittayakamee P Atsariyasing W Wannarit K Boonhong J Pongpirul K. The validity and reliability of the PHQ-9 in screening for post-stroke depression. BMC Psychiatry. (2020) 20:291. doi: 10.1186/s12888-020-02699-6, 32517743 PMC7285729

[20] Kroenke K Spitzer RL Williams JB. The PHQ-9: validity of a brief depression severity measure. J Gen Intern Med. (2001) 16:606–13. doi: 10.1046/j.1525-1497.2001.016009606.x, 11556941 PMC1495268

[21] de Ulíbarri JI González-Madroño A de Villar NG González P González B Mancha A . CONUT: a tool for controlling nutritional status. First validation in a hospital population. Nutr Hosp. (2005) 20:38–45. 15762418

[22] Puhr R Heinze G Nold M Lusa L Geroldinger A. Firth's logistic regression with rare events: accurate effect estimates and predictions? Stat Med. (2017) 36:2302–17. doi: 10.1002/sim.7273, 28295456

[23] Maldonado G Greenland S. Simulation study of confounder-selection strategies. Am J Epidemiol. (1993) 138:923–36. doi: 10.1093/oxfordjournals.aje.a116813, 8256780

[24] Bursac Z Gauss CH Williams DK Hosmer DW. Purposeful selection of variables in logistic regression. Source Code Biol Med. (2008) 3:17. doi: 10.1186/1751-0473-3-17, 19087314 PMC2633005

[25] Robinson RG Kubos KL Starr LB Rao K Price TR. Mood disorders in stroke patients. Importance Loc. Lesion Brain. (1984) 107:81–93. doi: 10.1093/brain/107.1.81, 6697163

[26] Khan M Ahmed B Ahmed M Najeeb M Raza E Khan F . Functional, cognitive and psychological outcomes, and recurrent vascular events in Pakistani stroke survivors: a cross sectional study. BMC Res Notes. (2012) 5:89. doi: 10.1186/1756-0500-5-89, 22321339 PMC3296616

[27] Wei N Yong W Li X Zhou Y Deng M Zhu H . Post-stroke depression and lesion location: a systematic review. J Neurol. (2015) 262:81–90. doi: 10.1007/s00415-014-7534-1, 25308633

[28] Mitchell AJ Sheth B Gill J Yadegarfar M Stubbs B Yadegarfar M . Prevalence and predictors of post-stroke mood disorders: a meta-analysis and meta-regression of depression, anxiety and adjustment disorder. Gen Hosp Psychiatry. (2017) 47:48–60. doi: 10.1016/j.genhosppsych.2017.04.001, 28807138

[29] Douven E Köhler S Rodriguez MMF Staals J Verhey FRJ Aalten P. Imaging markers of post-stroke depression and apathy: a systematic review and Meta-analysis. Neuropsychol Rev. (2017) 27:202–19. doi: 10.1007/s11065-017-9356-2, 28831649 PMC5613051

[30] Zabetian-Targhi F Srikanth VK Smith KJ Oddy DW Beare R Moran C . Associations between the dietary inflammatory index, brain volume, small vessel disease, and global cognitive function. J Acad Nutr Diet. (2021) 121:915–24. 33339764 10.1016/j.jand.2020.11.004

[31] Chander RJ Lam BYK Lin X Ng AYT Wong APL Mok VCT . Development and validation of a risk score (CHANGE) for cognitive impairment after ischemic stroke. Sci Rep. (2017) 7:12441. doi: 10.1038/s41598-017-12755-z, 28963553 PMC5622067

[32] Yan Q Liu M Xie Y Lin Y Fu P Pu Y . Kidney-brain axis in the pathogenesis of cognitive impairment. Neurobiol Dis. (2024) 200:106626. doi: 10.1016/j.nbd.2024.106626, 39122123

[33] Qiao Y Zhang Y Ding X Zhang Y Su X Zhang L . Sini decoction alleviates LPS-induced sepsis partly via restoration of metabolic impairments in the hypothalamic-pituitary-adrenal microenvironment. J Ethnopharmacol. (2025) 343:119456. doi: 10.1016/j.jep.2025.119456, 39922328

[34] Shirakabe A Hata N Kobayashi N Okazaki H Matsushita M Shibata Y . The prognostic impact of malnutrition in patients with severely decompensated acute heart failure, as assessed using the prognostic nutritional index (PNI) and controlling nutritional status (CONUT) score. Heart Vessel. (2018) 33:134–44. doi: 10.1007/s00380-017-1034-z, 28803356

[35] Gu M Wang J Xiao L Chen X Wang M Huang Q . Malnutrition and poststroke depression in patients with ischemic stroke. J Affect Disord. (2023) 334:113–20. doi: 10.1016/j.jad.2023.04.104, 37137412

[36] Shivappa N Steck SE Hurley TG Hussey JR Hébert JR. Designing and developing a literature-derived, population-based dietary inflammatory index. Public Health Nutr. (2014) 17:1689–96. doi: 10.1017/S1368980013002115, 23941862 PMC3925198

[37] David LA Maurice CF Carmody RN Gootenberg DB Button JE Wolfe BE . Diet rapidly and reproducibly alters the human gut microbiome. Nature. (2014) 505:559–63. doi: 10.1038/nature12820, 24336217 PMC3957428

[38] Zmora N Soffer E Elinav E. Transforming medicine with the microbiome. Sci Transl Med. (2019) 11:1815. doi: 10.1126/scitranslmed.aaw1815, 30700573

[39] Matijašić BB Obermajer T Lipoglavšek L Grabnar I Avguštin G Rogelj I. Association of dietary type with fecal microbiota in vegetarians and omnivores in Slovenia. Eur J Nutr. (2014) 53:1051–64. doi: 10.1007/s00394-013-0607-6, 24173964

[40] Kazemi A Noorbala AA Azam K Eskandari MH Djafarian K. Effect of probiotic and prebiotic vs placebo on psychological outcomes in patients with major depressive disorder: a randomized clinical trial. Clin Nutr. (2019) 38:522–8. doi: 10.1016/j.clnu.2018.04.010, 29731182

[41] Allen JM Berg Miller ME Pence BD Whitlock K Nehra V Gaskins HR . Voluntary and forced exercise differentially alters the gut microbiome in C57BL/6J mice. J Appl Physiol. (2015) 118:1059–66. doi: 10.1152/japplphysiol.01077.2014, 25678701

[42] Barnes JN. Exercise, cognitive function, and aging. Adv Physiol Educ. (2015) 39:55–62. doi: 10.1152/advan.00101.2014, 26031719 PMC4587595

[43] Loprinzi PD Edwards MK Crush E Ikuta T Del Arco A. Dose-response association between physical activity and cognitive function in a national sample of older adults. Am J Health Promot. (2018) 32:554–60. doi: 10.1177/0890117116689732, 29214828

