# Supplementary material for: Identifying key predictors of post-stroke depression and cognitive impairment in acute stroke survivors
Source: Front Neurol. 2026 May 20;17:1636511. doi: 10.3389/fneur.2026.1636511 (PMC13229621; doi:10.3389/fneur.2026.1636511)
Supplement: Supplementary file 1 [file Table_1.DOCX]

**Supplementary Material**

**Table S1 Comparison of clinical characteristics of the included patients**

| Variable | **Total（n=78）** | **Patients without PSD and PSCI（n=22）** | **Patients with PSCI/PSD（n=56）** | | | | **P** |
| --- | --- | --- | --- | --- | --- | --- | --- |
|  |  |  | **Patients with PSCI/PSD（n=56）** | **PSCI（n=13）** | **PSD（n=24）** | **PSCI and PSD**  **（n=19）** | |
| Age | 60.19 **±** 12.96 | 55.91 **±** 12.92 | 61.87 **±** 12.70 | 63.38 **±** 10.72 | 58.08 **±** 14.18 | 65.63 **±** 11.13 | 0.001* |
| Sex, male ,n（%） | 58（74.36） | 17（77.27） | 41（73.21） | 10（76.92,17.86） | 16（66.67,28.57） | 15（78.95,26.79 | 0.712 |
| Number of comorbidities | | |  |  |  |  | 0.034* |
| Hypertension, n（%） | 63（80.77） | 15（68.18） | 47（83.93） | 12（92.31,21.43） | 20（83.33,35.71） | 15（78.95,26.79） | |
| DM, n（%） | 34（43.60） | 9（40.91） | 25（44.64） | 4（30.77,7.14） | 9（37.50,16.07） | 12（63.16,21.43） | |
| Hyperlipidemia, n（%） | 39（50） | 10（45.45） | 29（51.78） | 4（30.77,7.14） | 13（54.17,23.21） | 12（63.16,21.43） | |
| CHD, n（%） | 10（12.82） | 0（.00） | 10（17.86） | 3（23.08,5.36） | 3（12.50,5.36） | 4（21.05,7.14） | |
| AF, n（%） | 7（8.97） | 0（.00） | 6（10.71） | 1（7.69,1.79） | 2（8.33,3.57） | 3（15.79,5.36） | |
| Smoke, n（%） | 37（47.44） | 12（54.55） | 25（44.64） | 6（46.15,10.71） | 11（45.83,19.64） | 8（42.11,14.29） | 0.432 |
| Drink, n（%） | 19（24.36） | 12（54.55） | 7（12.5） | 2（15.38,3.57） | 2（8.33,3.57） | 3（15.79,5.36） | 0.39 |
| Past history of stroke, n（%） | 14（17.95） | 1（4.55） | 13（23.21） | 4（30.77,7.14） | 3（12.50,5.36） | 6（31.58,10.71） | 0.007* |
| Regular exercise（%） | 41（52.56） | 14（63.64） | 27（48.21） | 10（76.92,17.86） | 12（50.00,21.43） | 5（26.31,8.93） | 0.025* |
| Right hemisphere lesion, n（%） | 50（64.10） | 11（50.00） | 39（69.64） | 11（84.62,19.64） | 18（75.00,32.14） | 10（52.63,17.86） | 0.042* |
| Large vessels’ stenosis, n（%） | 43（55.13） | 11（50.00） | 32（57.14） | 8（61.54,14.29） | 11（45.83,19.64） | 12（63.16,21.43） | 0.67 |

Note: Continuous data are presented as mean ± standard deviation (SD); categorical data are expressed as n (%).

Abbreviations: PSCI, post-stroke cognitive impairment; PSD, post-stroke depression; DM, diabetes mellitus; AF, atrial fibrillation; CHD, coronary heart disease.

Data interpretation: In the sub-columns under "Patients with PSCI/PSD (n = 56)", the two numbers in parentheses represent the percentage within that specific subgroup (e.g., n/13, n/13, n/24, or n/19) and the percentage within the total affected group (n/56), respectively.

Drink: Defined as regular alcohol consumption (more than 14 units per week for men or 7 units per week for women) prior to stroke onset.

Comorbidities: Defined as the presence of preexisting chronic conditions at the time of stroke onset. Key comorbidities assessed in this study included hypertension, DM, hyperlipidemia, CHD, AF

P-values: P-values were calculated using the Chi-square test or Fisher's exact test for categorical variables, and independent t-test or One-way ANOVA for continuous variables. **P* < 0.05 indicates statistical significance.

Table S2 Comparison of laboratory results of the included patients

| **Variable** | **total（n=78）** | **Patients without PSD and PSCI（n=22）** | **Patients with PSCI/PSD（n=56）** | | | | **P** |
| --- | --- | --- | --- | --- | --- | --- | --- |
|  |  |  | **Patients with PSCI/PSD（n=56）** | **PSCI（n=13）** | **PSD（n=24）** | **PSCI and PSD（n=19）** |  |
| WBC（10^9^/L） | 7.70 **±** 2.37 | 8.03 **±** 2.62 | 7.57 **±** 2.29 | 7.22 **±** 2.22 | 7.68 **±** 1.64 | 7.67 **±** 3.03 | 0.45 |
| Hb（g/L） | 142.07 **±** 16.36 | 142.95 **±** 17.24 | 141.73 **±** 16.16 | 143.39 **±** 11.40 | 141.92 **±** 14.50 | 140.37 **±** 20.95 | 0.79 |
| PLT（10^9^/L） | 208.14 **±** 60.65 | 228.48 **±** 58.62 | 200.52 **±** 60.13 | 195.15 **±** 46.56 | 207.58 **±** 58.81 | 195.26 **±** 71.18 | 0.001* |
| RDW | 13.36 **±** 2.15 | 12.64 **±** 2.12 | 13.624 **±** 2.11 | 13.61 **±** 2.06 | 13.62 **±** 2.08 | 13.64 **±** 2.30 | 0.001* |
| N（10^9^/L） | 5.03（3.65,6.24） | 4.96（3.77,7.8） | 5.09（3.52,6.08） | 4.52（3.34,7.59） | 5.28（4.28,6.14） | 4.98（3.39,5.81） | 0.491 |
| LYM（10^9^/L） | 1.56（1.18,1.83） | 1.61（1.18,1.84） | 1.56（1.18,1.83） | 1.55（1.12,1.7） | 1.64（1.38,2.01） | 1.48（1.28,1.79） | 0.985 |
| CRP (mg/L) | 3.445（2.53,6.81） | 3.85（2.6,6.62） | 3.37（2.39,7.19） | 3.37（2.0,6.26） | 3.05（1.48,5.64） | 3.77（3.00,29.66） | 0.415 |
| Pct (ng/ml) | 0.04（0.02,0.06） | 0.05（0.02,0.06） | 0.04（0.02,0.02） | 0.05（0.03,0.07） | 0.03（0.02,0.05） | 0.04（0.03,0.08） | 0.651 |
| Esr (mm/h) | 20（9,31） | 21（7,31） | 19.5（9.25,31） | 19（11,34） | 20（4.5,27.5） | 23（14,50.75） | 0.699 |
| INR | 0.96（0.93,0.99） | 0.96（0.93,1.02） | 0.96（0.90,0.99） | 0.98（0.91,1） | 0.95（0.89,0.98） | 0.99（0.92,1.01） | 0.41 |
| Serum Albumin（g/L） | 42（39,44） | 42（39,45） | 42（39.25,44） | 42（40.5,43.5） | 42（40,44） | 40（38,43） | 0.464 |
| AST, (U/L) | 24（19.5,31.5） | 23（18,29.5） | 25（20.25,33.25） | 19（16,28） | 28（22.25,39.25） | 26（21,35） | 0.129 |
| ALT, (U/L) | 24（22,28） | 23（21.5,26） | 25（22.25,29） | 25（21,33.25） | 25（22.25,28.75） | 25（22,29） | 0.159 |
| LDH | 172（146.5,215） | 165（143.5,186） | 178（148.25,326.25） | 179（137.5,247.5） | 188（148.2,379.5） | 176（164,207） | 0.037* |
| TBIL（μmol/L） | 11.5（8.75,14.9） | 10.3（8.5,12.9） | 11.75（8.88,14.9） | 10.3（8.05,14.95） | 12.3（8.8,15.43） | 11.7（10,14.9） | 0.373 |
| DBIL（μmol/L） | 2.58 **±** 1.85 | 1.86 **±** 1.15 | 2.86 **±** 2.00 | 2.73 **±** 2.14 | 2.97 **±** 2.12 | 2.80 **±** 1.83 | 0.037* |
| IBIL（μmol/L） | 8.7（6.25,11.5） | 8.6（6.05,11.4） | 9（6.325,11.83） | 9.4（5.45,12.35） | 8.7（7,10.98） | 8.9（5.3,12.35） | 0.507 |
| Cr（μmol/L） | 71.07 **±** 24.60 | 76.43 **±** 25.64 | 69.06 **±** 24.13 | 65.31 **±** 9.95 | 62.28 **±** 18.96 | 80.21 **±** 32.42 | 0.251 |
| BUN（mmol/L） | 5.38 **±** 1.46 | 4.70 **±** 0.90 | 5.636 **±** 1.56 | 5.92 **±** 1.36 | 5.08 **±** 1.16 | 6.15 **±** 1.92 | 0.012* |
| UA（μmol/L） | 348.26 **±** 106.07 | 359.10 **±** 99.80 | 344.2 **±** 108.92 | 308.38 **±** 75.06 | 325.12 **±** 87.36 | 392.79 **±** 137.25 | 0.582 |
| TC（mmol/L） | 4.68 **±** 1.03 | 4.80 **±** 1.01 | 4.63 **±** 1.05 | 4.36 **±** 0.9 | 4.70 **±** 0.82 | 4.72 **±** 1.35 | 0.514 |
| TG (mmol/L） | 1.56（1.08,2.175） | 1.69（1.14,3.65） | 1.47（0.93,1.88） | 1.35（0.83,1.63） | 1.56（1.13,1.86） | 1.63（0.93,2.85） | 0.153 |
| HDL-C（mmol/L） | 1.11 **±** 0.24 | 1.09 **±** 0.23 | 1.12 **±** 0.24 | 1.16 **±** 0.23 | 1.10 **±** 0.25 | 1.11 **±** 0.25 | 0.647 |
| LDL-C（mmol/L） | 2.91 **±** 0.94 | 2.85 **±** 0.85 | 2.93 **±** 0.98 | 2.77 **±** 0.93 | 2.93 **±** 0.87 | 3.05 **±** 1.16 | 0.73 |
| HCY（μmol/L） | 14.1（12,16.95） | 14.65（12.25,16.93） | 14.1（12,17.5） | 13.4（10.65,20） | 12.5（12.00,14.55） | 16.55（13.13,19.5） | 0.239 |
| FA | 9.55（6.28,14.78） | 8.2（5.73,11.63） | 10.85（6.48,16.75） | 10.95（5.75,20.63） | 11（6.3,16.6） | 10.7（9.3,17.4） | 0.001* |
| VB12（pg/ml） | 237（190,301） | 234.5（173,271） | 237.1（193,327.75） | 235.55（165,270.25） | 227（188,324） | 286.75（203,393.6） | 0.345 |

Notes: Continuous variables are presented as mean ± standard deviation (SD) for normally distributed data, or median (25th percentile, 75th percentile) for skewed data.

Abbreviations: WBC, white blood cell count; Hb, hemoglobin; PLT, platelet count; RDW, red cell distribution width; N, neutrophils; LYM, lymphocytes; CRP, C-reactive protein; PCT, procalcitonin; ESR, erythrocyte sedimentation rate; INR, international normalized ratio; ALB, albumin; AST, aspartate aminotransferase; ALT, alanine aminotransferase; LDH, lactate dehydrogenase; TBIL, total bilirubin; DBIL, direct bilirubin; IBIL, indirect bilirubin; Cr, creatinine; BUN, blood urea nitrogen; UA, uric acid; TC, total cholesterol; TG, triglyceride; HDL-C, high-density lipoprotein cholesterol; LDL-C, low-density lipoprotein cholesterol; HCY, homocysteine; FA, folic acid; VB12, vitamin B12.

Statistical Analysis: Inter-group comparisons across four clinical subgroups were performed using One-way ANOVA or the Kruskal-Wallis test. Comparisons between the total affected group (n=56) and the unaffected group (n=22) were performed using the independent t-test or Mann-Whitney U test. *$P < 0.05$ indicates statistical significance.

Table S3 Comparison of scale results of the included patients

| **variable** | **total（n=78）** | **Patients without PSD**  **and PSCI**  **（n=22）** | **Patients with PSCI/PSD（n=56）** | | | | **P** | |
| --- | --- | --- | --- | --- | --- | --- | --- | --- |
|  |  |  | **Patients with PSCI/PSD（n=56）** | **PSCI（n=13）** | **PSD（n=24）** | **PSCI and PSD**  **（n=19）** | |  |
| Education, (years) Median (IQR) | 12（9,15） | 12（8.75,15） | 12（9,15） | 10.5（8.25,14.25） | 12（9,16） | 9（8.25,12.75） | 0.787 | |
| First PHQ-9 | 3（1,7.5） | 1（0,1） | 5（2,10） | 1（0,2.75） | 6（4,10） | 9（4.25,13） | 0.001* | |
| First GAD-7 | 1（0,3） | 0（0,1） | 2（0,5.25） | 0（0,1） | 3（0.5,4.5） | 7（1,9） | 0.043* | |
| First MMSE | 27（25,29） | 28（26.5,30） | 26（25,29） | 25.5（22,26） | 28（26,30） | 25（19.5,28） | 0.045* | |
| NIHSS at admission | 3（1,5） | 2.5（1,3.25） | 3（1,5.75） | 3（1.5,8.5） | 3（1.25,5） | 2.5（1,9） | 0.244 | |
| mRS at admission | 1（1,1.5） | 1（1,2） | 1（1,3） | 1（1,3.5） | 1（1,3） | 1（1,2） | 0.24 | |
| ADL at admission | 20（14,34.75） | 14（14,25.5） | 22.5（16,35） | 21（14,39） | 28（16,35） | 20（16,35） | 0.11 | |
| AIS | 2（0,5） | 0（0,2.25） | 3（1,6） | 1.5（0,4.75） | 4（2,8） | 1.5（0,8.25） | 0.008 | |

Notes: Continuous data are presented as median (25th percentile, 75th percentile) due to non-normal distribution.

Abbreviations: PHQ-9, 9-item Patient Health Questionnaire (assessing depression severity); GAD-7, 7-item Generalized Anxiety Disorder scale (assessing anxiety severity); MMSE, Mini-Mental State Examination (assessing cognitive function); NIHSS, National Institutes of Health Stroke Scale (assessing neurological deficit severity); mRS, modified Rankin Scale (assessing functional disability/dependency); ADL, Activities of Daily Living (assessing physical functional capacity); AIS, Athens Insomnia Scale (assessing sleep quality).

Statistical Analysis: Inter-group comparisons across four clinical subgroups were performed using the Kruskal-Wallis test. Comparisons between the total affected group (n=56) and the unaffected group (n=22) were performed using the Mann-Whitney U test. *P < 0.05indicates statistical significance.

Table S4 Comparison of nutritional dietary structure of the included patient

| **variable** | **total（n=78）** | **without PSD and PSCI（n=22）** | **Patients with PSCI/PSD（n=56）** | | | | **P** |
| --- | --- | --- | --- | --- | --- | --- | --- |
|  |  |  | **Patients with PSCI/PSD（n=56）** | **PSCI（n=13）** | **PSD（n=24）** | **PSCI and PSD**  **（n=19）** |  |
| High-fat Diet, n（%） | 13（16.17） | 1（4.55） | 12（21.43） | 4（30.77,7.14） | 4（16.67,7.14） | 4（21.05,7.14） | 0.516 |
| High-fiber Diet, n（%） | 37（47.43） | 6（27.27） | 31（55.36） | 7 (53.85, 12.50) | 12 (50.00, 21.43) | 12 (63.16, 21.43) | 0.1 |
| CONUT | 1（1,2） | 1（1,2） | 2（1,2） | 2（1,3） | 1（0.25,2） | 2（1,2） | 0.168 |
| Nutritional Risk（according to CONUT）,n（%） | 37（47.44） | 6（27.27） | 31（55.36,39.74） | 9（69.23,16.07） | 9（37.5,16.07） | 13（68.42,23.21） | 0.029* |
| PNI | 43.23 **±** 3.70 | 43.43 **±** 4.6 | 43.16 **±** 3.35 | 44.00 **±** 2.27 | 43.33 **±** 2.93 | 42.37 **±** 4.32 | 0.776 |

Notes: Continuous variables are presented as mean ± standard deviation (SD) for normally distributed data (e.g., PNI), or as median (25th percentile, 75th percentile) for skewed data (e.g., CONUT). Categorical data are expressed as n (%).

Abbreviations: CONUT, Controlling Nutritional Status score; PNI, Prognostic Nutritional Index.

Definitions: 1. CONUT score assesses nutritional risk based on serum albumin level, total lymphocyte count, and total cholesterol level.

2. Nutritional Risk was defined as a total CONUT score >= 2 (covering mild, moderate, and severe malnutrition).

3. PNI was calculated using the formula: PNI = 10 × serum albumin (g/dL) + 0.005 × total lymphocyte count (per mm³)

Parentheses: In the sub-columns of "Patients with PSCI/PSD (n=56)", the two numbers in parentheses represent the percentage within that specific subgroup (n/13, n/24, or n/19) and the percentage within the total sample (n/78), respectively.

Statistical Analysis: $P$-values indicate the significance of the comparison between the total affected group (n=56) and the unaffected group (n=22). *$P < 0.05$ indicates statistical significance.
